# Metabolites of Procyanidins From *Litchi Chinensis* Pericarp With Xanthine Oxidase Inhibitory Effect and Antioxidant Activity

**DOI:** 10.3389/fnut.2021.676346

**Published:** 2021-09-21

**Authors:** Yong Sui, Jianbin Shi, Sha Cai, Tian Xiong, Bijun Xie, Zhida Sun, Xin Mei

**Affiliations:** ^1^Institute for Farm Products Processing and Nuclear-Agricultural Technology, Hubei Academy of Agricultural Science, Wuhan, China; ^2^College of Food Science and Technology, Huazhong Agricultural University, Wuhan, China

**Keywords:** litchi pericarp procyanidins, metabolite, phenolic acid, xanthine oxidase, antioxidant

## Abstract

Procyanidins from litchi pericarp (LPPC) has been evidenced to possess strong antioxidant activities *in vivo* that is possibly correlated with their intestinal metabolites. However, the xanthine oxidase inhibitory effect of LPPC and its metabolites was less concerned. In this study, three oligomeric procyanidins and eight metabolic phenolic acids were identified in the urine of rats administrated with LPPC by high performance liquid chromatography and liquid chromatography-mass spectrometry analysis. Data indicated that all the metabolites excreted were significantly increased by the treatment of 300 mg/kg body weight of LPPC (*P* < 0.05), revealing considerable 1, 1-Diphenyl-2-Picrylhydrazyl (DPPH) and hydroxyl radicals activities of scavenging. Moreover, phenolic metabolites involving epicatechin, A-type dimer, A-type trimer, caffeic acid, and shikimic acid exhibited greater xanthine oxidase inhibition effects compared with other metabolites, with an inhibitory rate higher than 50% at the concentration 200 μg/ml. The IC_50_ value of these five phenols were 58.43 ± 1.86, 68.37 ± 3.50, 74.87 ± 1.30, 95.67 ± 3.82, and 96.17 ± 1.64 μg/ml, respectively. As a whole, this work suggests that the xanthine oxidase inhibition and antioxidant activity of LPPC-derived metabolites as one of the mechanisms involved in the beneficial effects of LPPC against hyperuricemia or gout.

## Introduction

*Litchi chinensis* is widespread in the south of China, special taste and flavor making it popular in markets (1). As the inedible part of litchi, litchi pericarp (LPPC) is used as a traditional Chinese herbal medicine with hemostatic and acesodyne functions (2). Many studies have found that LPPC was rich in procyanidins, especially A-type procyanidins (3, 4). Various biological functions of A-type procyanidins in terms of their antioxidant activities have been reported, including scavenging-free radicals (5), decreasing serum uric acid (6), ameliorating atherosclerosis (7) and diabetic (8), as well as uropathogenic *Escherichia coli* antiadhesion activity (9). Moreover, oligomeric procyanidins were mainly absorbed in the small intestine and transferred to the liver, and those transferred procyanidins were hydrolyzed and degraded into phenolic acids in the colon due to the activity of enzymes of the colonic microflora (10, 11). The antioxidant capabilities of the A-type dimers were enhanced significantly after bioconversion by rat intestinal microbiota (12). The metabolic phenolic acids such as 4-hydroxyphenylpropionic acid and vanillic acid were able to inhibit the growth of several pathogenic intestinal bacteria and the cytokine-induced prostanoid biogenesis in human colonic fibroblasts, respectively (13, 14). These findings implied the bioactivities of A-type procyanidins that were related to its microbial-derived phenolic metabolites. However, there is still less attention to the xanthine oxidase inhibitory effect of metabolites of A-type procyanidins.

Gout is one of the most common metabolic disorders affecting more and more people because of the increasing incidence and severity of this disease over the last decades (15, 16). The disease is characterized by marked hyperuricemia, leading to the deposition of urate crystals in joints and kidneys, resulting in gouty arthritis and uric acid nephrolithiasis (17, 18). Serum uric acid is the end product of purine metabolism mediated by xanthine oxidase *in vivo*, which catalyzes the hydroxylation of hypoxanthine to xanthine and of xanthine to urate (19). Thus, one of the therapeutic approaches to treat gout is the use of xanthine oxidase inhibitors which prevent the production of uric acid. Allopurinol is the only xanthine oxidase inhibitor by prescription for the treatment of gout, but it is not the ideal drug because of some common side effects such as hepatitis, nephropathy, and allergic reactions (20, 21). Therefore, it is well-worth seeking effective xanthine oxidase inhibitors for the therapy of gout with no damage to the body.

Increasing researches demonstrated that natural products with excellent antioxidant capacity are the novel xanthine oxidase inhibitors, as xanthine oxidase generates hydrogen peroxide and might therefore be an initiator of tissue damage in a range of pathological states (22–24). Various polyphenolic compounds extracted from plants, especially flavonoids and procyanidins, have been evaluated previously for their inhibitory capacities against xanthine oxidase (6, 25–27). Moreover, several systemic *in vitro* screening studies on xanthine oxidase inhibitory activities of consumed beverages (28, 29) and medicinal plants used in traditional medicines around the world have been reported (30–35).

Thus, in this study, we identified and quantified the metabolites of LPPC procyanidins in the urine of rats by high performance liquid chromatography (HPLC) and liquid chromatography-mass spectrometry (LC-MS) analysis. Furthermore, the xanthine oxidase inhibitory effect and antioxidant activity of LPPC procyanidins and its metabolites were evaluated.

## Materials and Methods

### Chemicals and Materials

Fruit of litchi (*Litchi chinensis* Baila) was obtained from Guangzhou and arrived in the laboratory within 24 h of harvest. Fruits were peeled and the pericarp was quickly stored at −18°C until use.

β-glucuronidase Type H-5, (-)-epicatechin, vanillic acid, caffeic acid, *m*-coumaric acid, *p*-coumaric acid, 3-hydroxyphenylacetic acid, 3-hydroxyphenylpropionic acid, 4-hydroxyphenylpropionic acid, shikimic acid, xanthine oxidase, xanthine, and uric acid were obtained from Sigma (St. Louis, MO, USA). The β-glucuronidase demonstrated both β-glucuronidase (600 U/mg solid) and sulfatase (50 U/mg solid) activity. Grape seed procyanidin was purchased from Jianfeng Natural Product R&D Co., Ltd. (Tianjin, China). Acetonitrile and methanol (HPLC grade) were purchased from Fisher Scientific (Waltham, Massachusetts, USA). All the other chemicals and reagents were of analytical grade, unless specified.

### Isolation of Procyanidins From Litchi Pericarp

Procyanidins was separated from LPPC and purified using chromatography according to the method described previously (36). Fresh LPPC was torn into small fragments and extracted twice in a flask with 70% (v/v) aqueous ethanol at 50°C for 2 h. The extracts were collected and filtered under reduced pressure, and the filtrate was concentrated by rotatory evaporation at 40°C under vacuum to remove the ethanol. The crude procyanidins aqueous solution was applied to a column (300 × 30 mm, ID) packed with AB-8 macroporous resin (Nankai Hecheng Science & Technology, Tianjin, China). The column was rinsed with distilled water and eluted with 70% (v/v) aqueous ethanol at a flow rate of 10 ml/min. The eluent was concentrated under vacuum by rotatory evaporation at 40°C until the ethanol was completely removed and then freeze-dried. At the conclusion of this process, LPPC procyanidins was obtained.

### Butanol-HCl Assay

The procyanidin content of LPPC was determined using the butanol-HCl method reported by Porter et al. (37). The standard solution of different concentrations from 0 to 150 μg/ml was made using grape seed procyanidins. Measurement of each sample was performed in triplicate, and the procyanidin was detected at 546 nm.

### Animals

Male Sprague-Dawley rats (*n* = 20) were obtained at 6 weeks from Wuhan University Research Center for Animal Experiment, China. Animals were kept under a controlled temperature of 22 ± 1°C, relative humidity of 55–60%, and 12-h light/ 12-h dark cycle throughout the experiment. A normal solid diet (Wuhan University, Wuhan, China) and deionized water were available *ad libitum* for 1 week.

### Ethics Statement

All the experimental procedures involving animals followed the Guiding Principles in the Care and Use of Animals, and were approved by the ethics committee of Reference Laboratory for the test of Veterinary Drug Residues (SYXK, Hubei 2007-0044) and Huazhong Agricultural University, Hubei Province, China. We made all efforts to minimize suffering. The animals were killed by cervical dislocation under anesthesia.

### Analysis of Litchi Pericarp Procyanidins and Their Metabolites in Urine

Male Sprague-Dawley rats weighing 220 ± 20 g were randomly divided into two groups (*n* = 20) with 10 rats in each group. Prior to administration, animals were deprived of food for 12 h, but had access to deionized water. The LPPC was dissolved in physiological saline and administered orally to rats at a dose of 300 mg/kg body weight. In addition, the physiological saline was administered orally to the rats in another group as control. All the rats were placed in metabolic cages, each rat per cage (Jiayuan Technology Co. Ltd., Beijing, China). The samples of urine excreted from 0 to 24 h postadministration were collected from the bottom of the metabolic cage under chilled conditions using an ice bath and stored at −80°C before analysis, referring to the method described previously (38).

The sample of urine was centrifuged at 4,000 r/min for 10 min at 4°C, and the supernatant was collected. The supernatant was acidified to pH = 5.5 with 0.6 mol/l acetic acid and incubated at 37°C for 60 min in the presence of 10 KU β-glucuronidase with the activity of sulfatase. After further acidification to pH = 2 with 6 mol/l HCl, the samples were directly extracted by ethyl acetate × 3. The resulting supernatant was dried under vacuum by rotatory evaporation at 40°C until the ethyl acetate was completely removed and dissolved in 1 ml of 50% methanol. Then, the solution was purified by the solid phase extraction, and the residue was redissolved in methanol and analyzed by LC-MS as described by Li et al. with modifications (39).

Selected samples were analyzed on an Agilent 1100 LC-ESI-MS system (Agilent Technologies Co. Ltd., Foster City, California, USA) to identify metabolites in urine. A VP-ODS column (150 × 4.6 mm ID, 5 μm particle size, Shimadzu Co., Kyoto, Japan) was used and 20 μl solution of the sample was injected. The mobile phases consisted of 0.4% (v/v) aqueous acetic acid as A and acetonitrile as B with the following linear gradient: from 5 to 35% B in the first 40 min, from 35 to 50% B in the next 5 min, from 50 to 80% B in the next 5 min, and from 80 to 5% B in the next 5 min, and re-equilibrated with 5% B for 5 min before next injection. The flow rate was 0.2 ml/min and the absorbance of the eluate was monitored at 280 nm. Mass spectra were collected using an electrospray ionization (ESI) ion source in the negative mode under the following conditions: orifice voltage, −30 V; nebulizing pressure, 30 psi; heat capillary temperature, 275°C; and mass range, m/z 100-1,200.

### Xanthine Oxidase Inhibition Assay

To determine xanthine oxidase inhibitory activity, measuring the production of uric acid from xanthine substrate was the method of choice by most researchers and was chosen by us with slight modification (31, 40). The assay mixture consisted of 70 μl of 120 mmol/l phosphate buffer (pH = 7.5), 500 μl of 150 μmol/l xanthine (pH = 7.5), 400 μl of procyanidins, and solution of phenolic acids diluted to corresponding concentration in phosphate buffer and 30 μl of enzyme solution (0.5 units/ml in buffer). The reaction was initiated by the addition of enzyme and inhibition was evaluated after 2 min. Measurement of each sample was performed in triplicate and the absorbance was detected at 295 nm using a UV-1700 spectrophotometer (Shimadzu Co., Kyoto, Japan). Xanthine oxidase inhibitory activity was expressed as the percentage inhibition of xanthine oxidase in the aforementioned assay system, calculated according to the following equation:


$$\begin{array}{l} xanthine\;oxidase\;inhibition\;rate\;(\%)\;=\;\left[1-\frac{A_{i}-A_{j}}{A_{0}-A_{k}}\right]\times 100 \end{array}$$

where A_i_ and A_j_ were the activities of the test sample with and without xanthine oxidase, A_o_ was the activity of enzyme without test sample, and A_k_ was the control of A_o_ without test sample and enzyme. Allopurinol, a known inhibitor of xanthine oxidase, was used as a positive control. IC_50_ values were calculated from the mean values of data.

### 1, 1-Diphenyl-2-picrylhydrazyl Radical Scavenging Activity

1, 1-Diphenyl-2-picrylhydrazyl radical scavenging activity was determined referring to the method described previously (41). Litchi pericarp and phenolic acids were dissolved in 1.0 ml of deionized water at various concentrations ranging from 2.0 to 100.0 μg/ml. Then, the solution of the sample was mixed with 1.0 ml of freshly prepared 0.2 mM DPPH ethanolic solution. Control in the experiment was prepared by the same reagent without the sample. All the tubes were incubated at 37°C for 30 min in the dark, and the final solution was recorded at 517 nm. The DPPH radical scavenging activity was calculated using the following equation:


$$\begin{array}{l} DPPH\;radical\;scavenging\;rate\;\left(\%\right)\\ \;=\frac{\left(A_{control}-A_{sample}\right)}{A_{control}}\;\times \;100 \end{array}$$

### Hydroxyl Radical Scavenging Activity

Hydroxyl radical scavenging activity was assessed according to the method of Dong et al. (42) with modifications. The reagents and samples were added strictly obeying following order: 0.25 ml 1.5 mM phenanthroline ethanolic solution, 0.5 ml phosphoric acid buffer (pH = 7.4), 0.25 ml 1.5 mM freshly prepared ferrous sulfate solution, 0.5 ml sample (0–0.5 mg), and 0.25 ml 0.01% H_2_O_2_. The injured tubes in the experiment were prepared by the same reagents except samples, while control was carried out by adding sample without H_2_O_2_. The mixtures were shaken and incubated at 37°C for 1 h, and then measured at 536 nm. The hydroxyl radical scavenging activity was calculated using the following equation:


$$\begin{array}{l} hydroxyl\;radical\;scavenging\;rate\;\left(\%\right)\\ \;=\frac{A_{sample}-A_{injured}}{A_{control}-A_{injured}}\;\times 100 \end{array}$$

### Statistical Analysis

Data were expressed as mean ± SD. All data were analyzed using a one-way ANOVA, followed by Duncan *post-hoc* test if the difference was significant (*P* < 0.05) in groups. Statistical analyses were conducted by the SPSS 16.0 software (International Business Machines, Corp., Armonk, USA), and a difference was regarded as significant when *P* < 0.05.

## Result and Discussion

### Analysis of Procyanidin Content in the LPPC

Litchi pericarp gave a positive reaction with butanol-HCl assay, indicating the presence of abundant procyanidins. The content of procyanidins in LPPC was 99.24 ± 2.01% compared with grape seed procyanidins, which were used as a standard substance. The content of procyanidins in grape seed was 95.00%.

### Determination of A-Type Procyanidins and Their Metabolites in Urine of Rat

During the whole experiment, there was no significant difference between control rats and the rats administrated with 300 mg/kg LPPC in body weight, intake of food, and intake of water (Supplementary Table 1).

**Figure 1:**
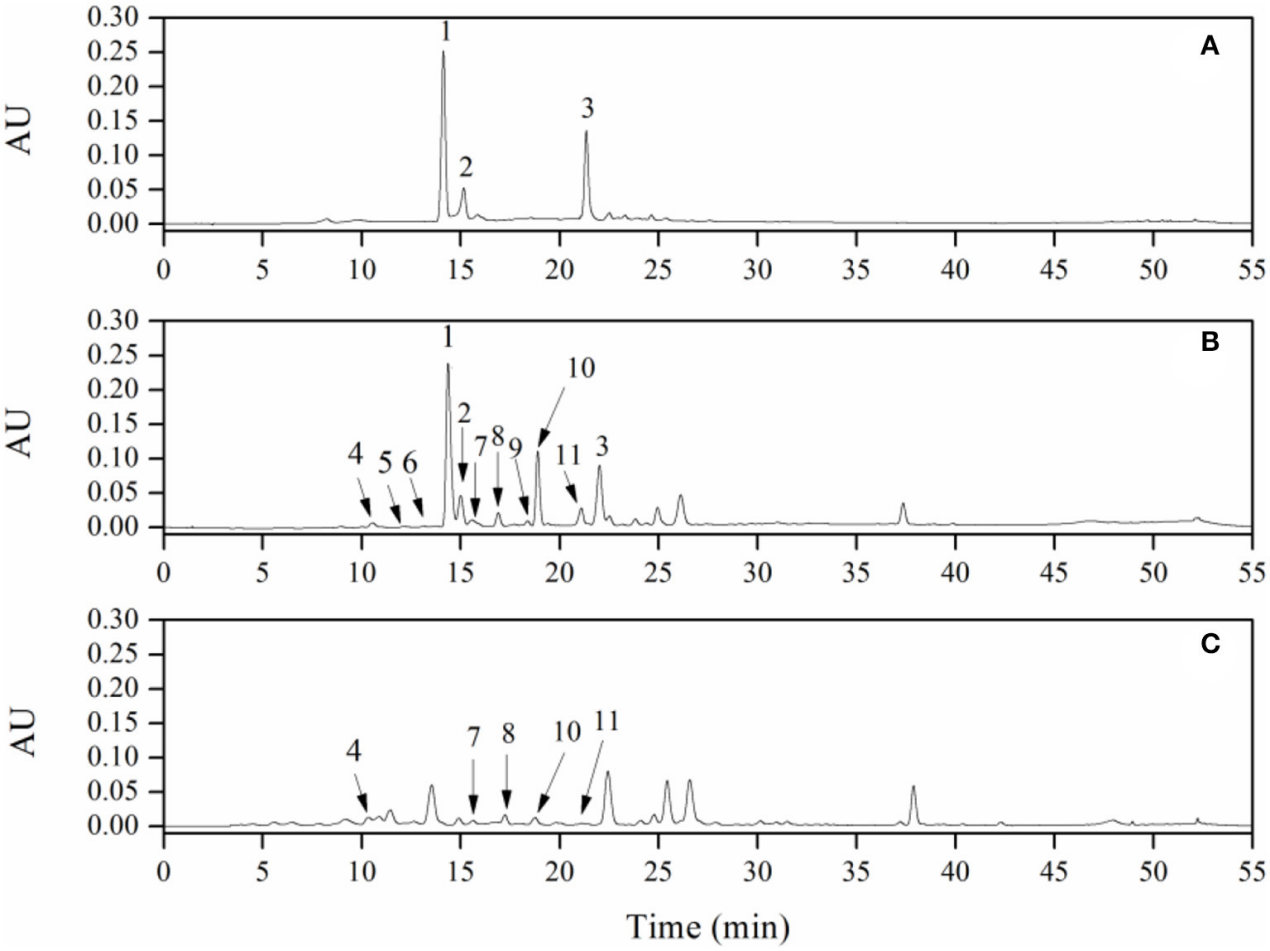
RP-HPLC chromatogram of litchi pericarp (LPPC) (**A**, 1 mg/ml) and their urinary metabolites in rats: **(B)** of experimental group with LPPC administration dose of 300 mg/kg·bw, **(C)** of control group (1, (-)-Epicatechin; 2, A-type trimer; 3, A-type dimer; 4, 3-hydroxyphenylacetic acid; 5, vanillic acid; 6, caffeic acid; 7, 3-hydroxyphenylpropionic; 8, *p*-coumaric acid; 9, shikimic acid; 10, 4-hydroxyphenylpropionic acid; and 11, *m*-coumaric acid).

Major metabolites of LPPC in the urine of rat were analyzed after deconjugation by glucuronidase/sulfatase using HPLC-ESI-MS/MS and compared with the authentic reference (Table 1). There were three main peaks in the chromatogram of RP-HPLC analysis of LPPC (Figure 1A). Peak 1 had a single molecular ion [M-H]^−^ at m/z 289.1 and with major fragment ions at m/z 245.0 and 205.0. Based on the mass spectrum and comparison with an authentic standard, this compound was identified as (-)-epicatechin. Peak 2 and Peak 3 owned one molecular ion peak [M-H]^−^ at m/z 863.5 and 575.2, respectively. The molecular ion [M-H]^−^ at m/z 863.5, which produced MS^2^ fragment ion at m/z 711.1, 693.2, 573.0, 451.0, and 289.0, was similar to A-type procyanidin trimer. The molecular ion [M-H]^−^ at m/z 575.2 with MS^2^ major fragments at m/z 449.0, 423.0, 289.0, and 285.0, was identified as A-type procyanidin dimer (36). In addition to the aforementioned procyanidins, there were eight metabolic phenolic acids identified in the urine of the rats with 300 mg/kg LPPC (Figure 1B). The phenolic acids selected for analysis were those reported previously as microbial metabolites of procyanidins *in vitro* and *in vivo* (39).

**Table 1:** Metabolites excreted in urine within 24 h fed with litchi pericarp (LPPC).

**Compounds**	**Retention time (min)**	**Parent ion (m/z)**	**Product ion (m/z)**	**Urinary excretion**	
				**Control**	**300 mg/kg**
**Procyanidin (% to oral dose)**					
(-)-Epicatechin	14.7	289.1	245.0, 205.0	ND	0.21 ± 0.04^*^
A-type trimer	15.8	863.5	711.1, 693.2, 573.0, 451.0, 289.0	ND	0.43 ± 0.02^*^
A-type dimer	22.1	575.2	449.0, 423.0, 289.0, 285.0	ND	0.54 ± 0.09^*^
**Phenolic acids (nmol)**					
3-Hydroxyphenylacetic acid	11.5	151.1	107.0 (CO_2_ loss)	13.99 ± 1.24	46.98 ± 3.76^*^
Vanillic acid	12.6	167.0	123.0 (CO_2_ loss)	ND	6.62 ± 2.79^*^
Caffeic acid	13.4	179.1	135.0 (CO_2_ loss)	ND	20.67 ± 2.68^*^
3-Hydroxyphenylpropionic acid	16.6	165.5	121.1 (CO_2_ loss)	6.38 ± 0.74	12.87 ± 3.06^*^
*p*-Coumaric acid	17.1	163.1	118.8 (CO_2_ loss)	7.15 ± 1.37	13.89 ± 1.65^*^
Shikimic acid	18.0	173.2	111.2 (CO_2_ + H_2_O loss)	ND	27.22 ± 7.02^*^
4-Hydroxyphenylpropionic acid	18.5	165.5	121.1 (CO_2_ loss)	5.75 ± 1.99	60.54 ± 3.18^*^
*m*-Coumaric acid	21.2	163.1	118.8 (CO_2_ loss)	2.69 ± 0.17	39.01 ± 7.33^*^

* *Indicated the significant differences (P < 0.05) between the two groups*.

**Figure 2:**
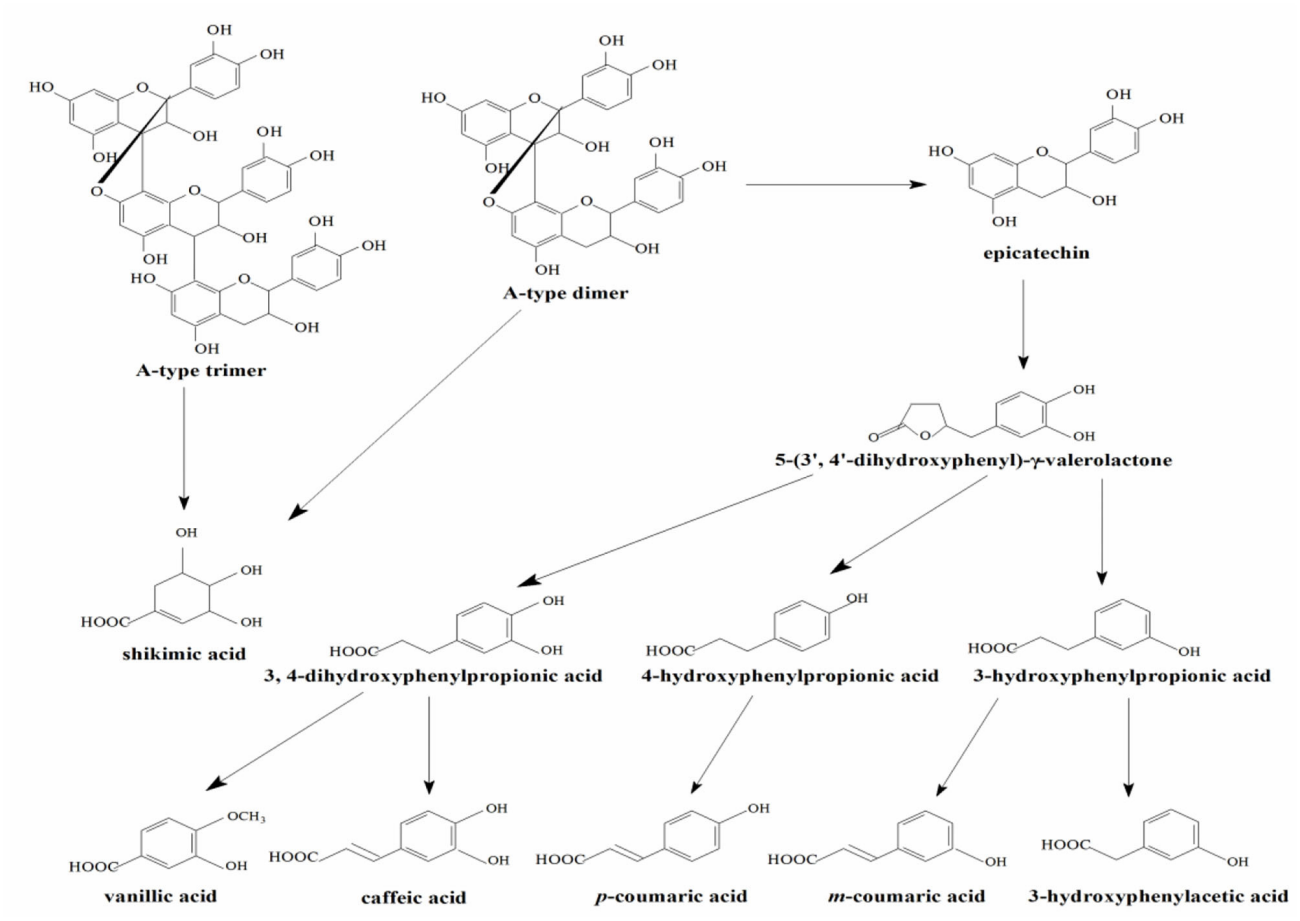
Proposed metabolic pathway of LPPC by the intestinal microflora.

In the experimental group of LPPC, abundant procyanidins and phenolic acids were observed in the urine of rats. The results showed that the LPPC was absorbed and translated in rats. The total urinary excretion of (-)-epicatechin accounted for 0.21 ± 0.04% (mol/mol) of the oral intake. The content of A-type procyanidin dimer and trimer excreted in the urine was also low, namely, 0.54 ± 0.09% (mol/mol) and 0.43 ± 0.02% (mol/mol) compared with the administration sample at dose of 300 mg/kg·bw, respectively. The polarity of A-type procyanidin dimer was weaker than (-)-epicatechin and A-type procyanidin trimer based on their retention time in RP-HPLC analysis. Therefore, the amount of A-type procyanidin dimer was the largest in urine, suggesting that it was less absorbed than (-)-epicatechin and A-type procyanidin trimer in the rats. The recovery rates of procyanidins *in vivo* indicated that their absorption and excretion in rats were affected by the structure and polarity. As shown in Table 1, 3-hydroxyphenylacetic acid, 3-hydroxyphenylpropionic acid, 4-hydroxyphenylpropionic acid, *p*-coumaric acid, and *m*-coumaric acid were detected in both control (Figure 1C) and treatment samples of urine, while vanillic acid, caffeic acid, and shikimic acid were exclusively found in the urine of rats administered with LPPC. However, the levels of all metabolic phenolic acids excreted in the urine were very significantly increased by 300 mg/kg body weight of LPPC (*P* < 0.05). Furthermore, 3-hydroxyphenylacetic acid, shikimic acid, 4-hydroxyphenylpropionic acid, and *m*-coumaric acid accounted for more than 76% of the total metabolic phenolic acids. The fragmentation pathway of these metabolic phenolic acids was shown in Figure 2 (43, 44).

Recently, increasing studies demonstrated the biological functions of the metabolic phenolic acids of procyanidins. Wu et al. reported that metabolites of procyanidin oligomers from lotus seedpod such as ferulic acid and syringic acid had better antiglycative activities and might inhibit the formation of advanced glycation end products (45). Hippuric acid, alpha-hydroxyhippuric acid, and 3,4-dihydroxyphenylacetic acid, which were detected in A-type procyanidins from cranberry, exhibited effective antiadhesive activity (9). Some phenolic acids, including 4-hydroxyphenylpropionic acid, 4-hydroxyphenylacetic acid, gallic acid, and caffeic acid derived from the microbial degradation of tea catechins were able to inhibit the growth of several pathogenic and non-beneficial intestinal bacteria without significantly affecting the growth of beneficial bacteria (*Lactobacillus* spp. and *Bifidobacterium* spp.) (13). The analysis of metabolic phenolic acids and oligomeric procyanidins in the urine of rat in this study, to some extent, will help to explain the benefits of procyanidins in the health of human.

### Xanthine Oxidase Inhibition of LPPC and Its Metabolites

As shown in Table 2, LPPC, epicatechin, A-type dimer, A-type trimer, and eight metabolic phenolic acids (12 samples) demonstrated xanthine oxidase inhibitory activity at the concentration of 200 μg/ml, among which eight samples showed an inhibition higher than 50%. The most active samples were epicatechin, A-type dimer, and A-type trimer, exhibiting as low IC_50_ as 58.43 ± 1.86, 68.37 ± 3.50, and 74.87 ± 1.30 μg/ml, respectively, followed by samples of LPPC, caffeic acid, and shikimic acid, with IC_50_ = 79.77 ± 1.80, 95.67 ± 3.82, and 96.17 ± 1.64 μg/ml, respectively. Furthermore, *p*-coumaric acid and *m*-coumaric acid processed considerable xanthine oxidase inhibition effects, as well. However, 3-hydroxyphenylacetic acid, 3-hydroxyphenylpropionic acid, 4-hydroxyphenylpropionic acid, and vanillic acid exhibited slight inhibitory effects, with IC_50_ > 200 μg/ml. As the standard drug for gout, allopurinol demonstrated excellent xanthine oxidase inhibitory activity, which showed 85.60 ± 3.13% inhibition at 200 μg/ml with IC_50_ = 5.63 ± 0.45 μg/ml.

**Table 2:** Xanthine oxidase inhibitory properties of LPPC and its metabolites.

**Compounds**	**Inhibition (%) at 200 μg/ml**	**IC_**50**_ (μg/ml)**
LPPC	64.67 ± 1.62^e^	79.77 ± 1.80^e^
(-)-Epicatechin	78.63 ± 1.72^b^	58.43 ± 1.86^b^
A-type dimer	71.37 ± 2.32^c^	68.37 ± 3.50^c^
A-type trimer	67.80 ± 0.40^d^	74.87 ± 1.30^d^
3-Hydroxyphenylacetic acid	21.13 ± 1.78^k^	>200
3-Hydroxyphenylpropionic acid	27.03 ± 1.44^ij^	>200
4-Hydroxyphenylpropionic acid	29.43 ± 0.75^i^	>200
Vanillic acid	25.03 ± 1.47^j^	>200
Shikimic acid	61.13 ± 0.81^f^	96.17 ± 1.64^f^
Caffeic acid	60.67 ± 2.16^f^	95.67 ± 3.82^f^
*p*-Coumaric acid	57.40 ± 0.62^g^	139.90 ± 5.80^g^
*m*-Coumaric acid	52.80 ± 1.57^h^	154.57 ± 5.70^h^
Allopurinol	85.60 ± 3.13^a^	5.63 ± 0.45^a^

*The means in the same column sharing a different letter were significantly different (P < 0.05) between the groups*.

Numerous clinical trials have reported that xanthine oxidase is an important enzyme in radical-mediated diseases (46, 47). Because of multiple phenolic hydroxyl groups, procyanidins might provide a remedy in radical-mediated conditions at various stages of oxidative stress (48). Proanthocyanidins from litchi fruit significantly decreased serum concentration of uric acid in healthy male volunteers and inhibited xanthine oxidase activity in a dose-dependent manner *in vitro* experiment (6). Phenolic acids such as caffeic acid and *p*-coumaric acid, were reported to act as potent xanthine oxidase inhibitors, and the hydrogen bonding and hydrophobic interactions were the main forces in the binding of phenolics to xanthine oxidase (49, 50). Because of the stronger electrostatic force with xanthine oxidase, flavonoids exhibited higher xanthine oxidase inhibitory capacity as compared with derivatives of gallic acid (22). Similar to the published data, epicatechin, A-type dimer, A-type trimer, and several metabolites of LPPC (caffeic acid, shikimic acid, and *p*-coumaric acid) possessed higher inhibitory activity toward xanthine oxidase in this study.

### Antioxidant Activity of LPPC and Its Metabolites

As shown in Table 3, LPPC, epicatechin, A-type dimer, A-type trimer, and eight metabolic phenolic acids exhibited different radical scavenging activities. In DPPH radical scavenging assay, epicatechin and A-type dimer demonstrated the strongest activity among all of the metabolites, with IC_50_ = 5.43 ± 0.10 and 5.86 ± 0.14 μg/ml, respectively. A-type trimer, shikimic acid, LPPC, caffeic acid, *p*-coumaric acid, and *m*-coumaric acid also processed considerable DPPH radical scavenging activity, with IC_50_ = 8.00 ± 0.18, 8.96 ± 0.18, 9.34 ± 0.16, 9.88 ± 0.55, 16.53 ± 0.65, and 21.93 ± 1.25 μg/ml, respectively. However, 3-hydroxyphenylacetic acid, 3-hydroxyphenylpropionic acid, 4-hydroxyphenylpropionic acid, and vanillic acid showed significantly weaker activity, with IC_50_ > 50 μg/ml.

**Table 3:** Antioxidant activity of LPPC and its metabolites in different assays.

**Compounds**	**IC**_**50**_ **(μg/ml)**	
	**DPPH·**	**·OH**
LPPC	9.34 ± 0.16^bc^	143.47 ± 3.26^b^
(-)-Epicatechin	5.43 ± 0.10^a^	121.27 ± 1.80^a^
A-type dimer	5.86 ± 0.14^a^	126.57 ± 2.58^a^
A-type trimer	8.00 ± 0.18^b^	122.73 ± 2.35^a^
3-Hydroxyphenylacetic acid	82.60 ± 1.95^h^	803.47 ± 6.38^g^
3-Hydroxyphenylpropionic acid	77.83 ± 0.93^g^	841.57 ± 4.45^h^
4-Hydroxyphenylpropionic acid	74.63 ± 1.40^f^	813.87 ± 5.48^g^
Vanillic acid	86.06 ± 1.33^i^	897.33 ± 9.90^i^
Shikimic acid	8.96 ± 0.18b^c^	186.53 ± 7.56^d^
Caffeic acid	9.88 ± 0.55^c^	161.47 ± 3.31^c^
*p*-Coumaric acid	16.53 ± 0.65^d^	220.13 ± 4.57^e^
*m*-Coumaric acid	21.93 ± 1.25^e^	256.43 ± 9.33^f^

*The means in the same column sharing a different letter were significantly different (P < 0.05) between the groups*.

Hydroxyl radicals were known to be the most reactive of all the reactive oxygen species and were thought to initiate cell damage *in vivo* (2). In hydroxyl radical scavenging assay, the results were similar to those of DPPH scavenging activity. The hydroxyl radical scavenging activities of oligomeric procyanidins were significantly stronger than metabolic phenolic acids (*P* < 0.05). Among all of the phenolic acids, caffeic acid possessed the highest activity while vanillic acid showed the lowest, with IC_50_ = 161.47 ± 3.31 and 897.33 ± 9.90 μg/ml, respectively. The hydroxyl radical scavenging activities of metabolic phenolic acids were as follows: caffeic acid > shikimic acid > *p*-coumaric acid > *m*-coumaric acid > 3-hydroxyphenylacetic acid > 4-hydroxyphenylpropionic acid > 3-hydroxyphenylpropionic acid > vanillic acid.

The radical scavenging effects of polyphenols depended on the number of hydroxyl groups in their molecular structures (48). Oligomeric procyanidins, A-type dimer, and the trimeric procyanidins had a strong scavenging effect on hydroxyl radical, and the antioxidant activities of them seemed to be related to the number of hydroxyls in their molecular structures (3). Lotus seedpod oligomeric procyanidins, catechin, and caffeic acid had greater antioxidant capacities than *m*-coumaric acid 3-hydroxyphenylacetic acid, and 3-hydroxyphenylpropionic (45). Similar to this study, multiple phenolic hydroxyl groups of procyanidins produced better antioxidant activities compared with metabolic phenolic acids. Because of the more hydroxyl groups in structures, caffeic acid and shikimic acid demonstrated higher radicals scavenging activities compared with other phenolic acids. The results also showed that the xanthine oxidase inhibitory activities of metabolites were positively correlated with their antioxidant activities. Therefore, the excellent xanthine oxidase inhibitory effects of oligomeric procyanidins, caffeic acid, and shikimic acid were probably due to their better antioxidant activities.

Although an enormous amount of clinical trials suggested that polyphenols improve radical-mediated diseases, the inconsistency of some research triggered an interest in interindividual variability in response to consumption of polyphenols. The intestinal microbiota might be the potential target, since there were variations in intestinal microbiota in different individuals and affected the bioavailability of polyphenols (51). Numerous studies demonstrated that the antihyperuricemia activities of polyphenol were related to regulating intestinal microbiota; in turn, the intestinal microbiota influence the metabolism of polyphenol (10, 52). In this study, oligomeric procyanidins and several metabolites of LPPC (caffeic acid, shikimic acid, and *p*-coumaric acid) possessed high-inhibitory activity toward xanthine oxidase and strong antioxidant activity. Some limitations also exist in this paper. It is necessary to compare the metabolites of LPPC and intestinal microbiota in normal and hyperuricemia model of rat, which will help to explain the antihyperuricemia mechanism of LPPC. Furthermore, future research might also focus on specific group of intestinal microbiota in regulating metabolism of procyanidins to better understand its health benefits in persons.

## Conclusions

In conclusion, three oligomeric procyanidins and eight metabolic phenolic acids were identified in the urine of the rats administrated with LPPC by LC-MS. The xanthine oxidase inhibitory effect and antioxidant activity of those metabolites were also investigated in this study. It was demonstrated that epicatechin, A-type dimer, A-type trimer, caffeic acid, and shikimic acid processed better xanthine oxidase inhibition effects and radicals scavenging properties than other metabolites. Moreover, the xanthine oxidase inhibitory activities of the metabolites were correlated positively with their antioxidant activities. It is supposed that LPPC and its metabolites may provide significant inhibitory effects on the formation of uric acid *in vivo* and be useful for the treatment of hyperuricemia.

## Data Availability Statement

The raw data supporting the conclusions of this article will be made available by the authors, without undue reservation.

## Ethics Statement

The animal study was reviewed and approved by Reference Laboratory for the test of Veterinary Drug Residues.

## Author Contributions

YS helped in performing the experiments, writing the original draft, and editing the manuscript. JS, SC, and TX helped perform the experiments and revising the manuscript. BX and ZS contributed in conceiving ideas, conceptualization, and methodology. XM contributed writing—reviewing and editing. All authors contributed to the article and approved the submitted version.

## Funding

This work was supported by the National Key Technologies Program of China during the 12th Five Year Plan Period (2012BAD31B03), Youth Foundation of Hubei Academy of Agricultural Science (2018NKYJJ16), and the Key Laboratory of Deep Processing of Major Grain and Oil (Ministry of Education, 2018JYBQGDKFA03).

## Conflict of Interest

The authors declare that the research was conducted in the absence of any commercial or financial relationships that could be construed as a potential conflict of interest.

## Publisher's Note

All claims expressed in this article are solely those of the authors and do not necessarily represent those of their affiliated organizations, or those of the publisher, the editors and the reviewers. Any product that may be evaluated in this article, or claim that may be made by its manufacturer, is not guaranteed or endorsed by the publisher.
