# Supplementary material for: Metabolites of Procyanidins From Litchi Chinensis Pericarp With Xanthine Oxidase Inhibitory Effect and Antioxidant Activity
Source: Front Nutr. 2021 Sep 21;8:676346. doi: 10.3389/fnut.2021.676346 (PMC8490629; doi:10.3389/fnut.2021.676346)
Supplement: Supplementary file 1 [file Table_1.pdf]

SUPPLEMENTARY MATERIALS

TABLE S1 The body weight, food intake and water intake during the whole experiment in rats

	**Groups**	
	**Control**	**300 mg/kg**
Initial body weight (g)	218.05±10.97	216.79±12.67
Final body weight (g)	259.42±15.21	257.07±16.42
Food intake (g per day)	18.89±1.69	19.20±2.31
Water intake (g per day)	25.75±3.67	25.32±2.48
